# Hypoxic-Ischemic Neonatal Encephalopathy: Animal Experiments for Neuroprotective Therapies

**DOI:** 10.1155/2013/659374

**Published:** 2013-02-27

**Authors:** Hiroshi Sameshima, Tsuyomu Ikenoue

**Affiliations:** Department of Obstetrics and Gynecology and Center for Perinatal Medicine, Faculty of Medicine, University of Miyazaki, 5200 Kiyotake, Kihara, Miyazaki 889-1692, Japan

## Abstract

Hypoxic-ischemic neonatal encephalopathy and ensuing brain damage is still an important problem in modern perinatal medicine. In this paper, we would like to share some of the results of our recent studies on neuroprotective therapies in animal experiments, as well as some literature reviews. From the basic animal studies, we have now obtained some possible candidates for therapeutic measures against hypoxic-ischemic neonatal encephalopathy. For example, they are hypothermia, rehabilitation, free radical scavenger, neurotrophic factors and growth factors, steroid, calcium channel blocker, vagal stimulation, some anti apoptotic agents, pre- and post conditioning, antioxidants, cell therapy with stem cells, modulators of K(+)-ATP channels, and so on. Whether combination of these therapies may be more beneficial than any single therapy needs to be clarified. Hypoxia-ischemia is a complicated condition, in which the cause, severity, and time-course are different in each case. Likewise, each fetus has its own inherent potentials such as adaptation, preconditioning-tolerance, and intolerance. Therefore, further extensive studies are required to establish an individualized strategy for neuroprotection against perinatal hypoxic-ischemic insult.

## 1. Introduction

Hypoxic-ischemic brain damage caused by intrapartum disastrous events is still an important problem in modern obstetrics even in developed countries. It accounts for 10% to 20% of infants with cerebral palsy [1, 2].

Since 1997, we have been performing a regional population-based study on intrauterine fetal deaths, neonatal deaths, and severely handicapped infants [1]. From a total of 140,000 deliveries in the last 13 years, we found a perinatal mortality rate of 4 per 1,000. This is the lowest rate in the world (perinatal mortality includes stillbirths ≥22 weeks of gestation and neonatal deaths ≤7 days of life). However, even where the most advanced perinatal services are available, the incidence of brain damage is 2/1,000, similar to rates around the world [2]. Among infants with brain damage, the most frequent cause is congenital abnormality (1/3), and hypoxic-ischemic encephalopathy constitutes 15%.

Thus, it is important for us to study (1) how to predict fetal hypoxic-ischemic events early enough to prevent brain damage, (2) how to treat severely damaged neonates immediately after birth to prevent brain damage, and (3) how to individualize fetuses at high-risk of brain damage?

We have been performing clinical and basic animal studies to elucidate the pathogenesis of hypoxic-ischemic brain damage of neonates. In this context, we have also performed animal studies to seek neuroprotective therapies against hypoxia-ischemia. In this paper, we would like to show some of the results of our recent studies on neuroprotective therapies in animal experiments, as well as some literature reviews on neuroprotective therapies.

## 2. The Levine-Rice Model

We have been using the Levine-Rice model to study neonatal hypoxic-ischemic brain damage. This model has been widely used for 3 decades for histological analysis as well as behavioral tests.

### 2.1. Preparation of the Model

The hypoxic-ischemic encephalopathy model in adult can be done in a variety of ways. One method involves introducing a ligation the unilateral carotid artery and exposing it to whole-body hypoxia [3]. This Levine preparation was modified for neonatal rats, for example, in order to examine birth asphyxia [4]. In employing the Levine-Rice model to study perinatal hypoxic-ischemic encephalopathy, we used 7-day-old Wistar rats because the developmental maturity of their brains roughly corresponds to that of near-term or term fetal brain in human beings [4].

**Figure 1:**
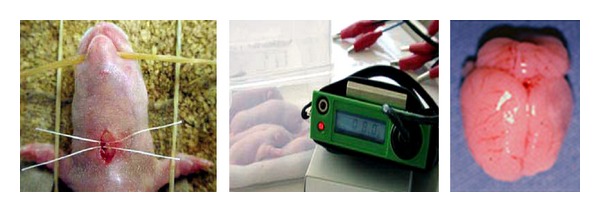
The Levine-Rice model of 7-day-old Wistar rat (from left to right). Under ether anesthesia, skin was incised, and unilateral common carotid artery was doubly ligated. After recovery, they were transferred to a chamber containing humidified 8% hypoxic gas. Brain was removed for histological study. The ligated side of the hemisphere was atrophic.

The Levine-Rice model was made as follows [5] (Figure 1). The 7-day-old Wistar rat was lightly anesthetized by ether inhalation, and the left carotid artery was sectioned between a double ligature with 4–0 surgical silk. The rat was allowed to recover for 2 hours or more and then exposed to 8% hypoxia, by being placed in a hypoxic chamber at 32 degrees Celsius, which is the usual ambient temperature of the neonatal rat. After hypoxia, the rats were removed from the chamber and returned to their dams.

### 2.2. Histological Grading of Severity of Brain Damage

**Figure 2:**
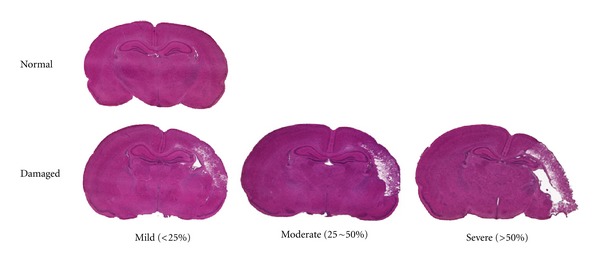
Severity of brain damage was classified into normal, mild, moderate, or severe according to the damaged area.

In this model, the ligated side of the brain hemisphere is exposed to hypoxia and hypoperfusion (hypoxia-ischemia) and the nonligated side is exposed to hypoxia only. From a histological standpoint, the nonligated side has long been used as the control and the ligated side is used as the experimental side. Severity of brain damage was graded into 4 categories: normal (no damage), mild (<25%), moderate (25–50%), and severe (>50%) of the surface area on a single section with neuronal loss [5] (Figure 2).

### 2.3. Blood Flow Distribution to the Brain Hemispheres

**Figure 3:**
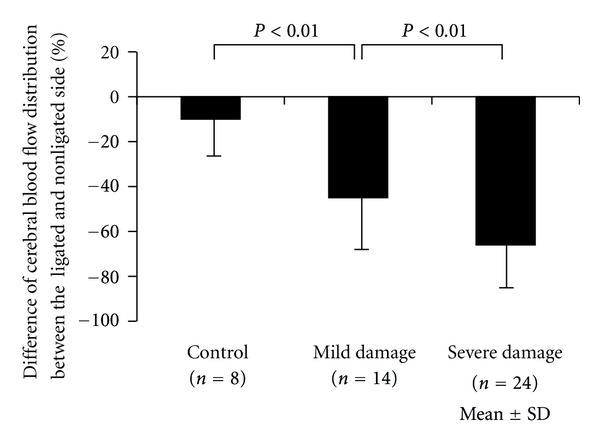
Percent difference of cerebral blood flow distribution between the ligated and nonligated side was expressed. The more severely damaged, the less blood flow distributed to the damaged brain hemisphere. Bars represent mean ± SD.

As mentioned, this model causes hypoperfusion in the ligated side of the brain, while the nonligated side is exposed to hypoxia alone. Wistar rats have anatomical arterial connections between the right and left side of the brain hemispheres. With a radioactive tracer technique, regional cerebral blood flow was decreased in the ligated side following carotid artery ligation and hypoxia [6]. We used a colored microsphere technique and investigated cerebral blood flow distribution and the resulting grade of hypoxic-ischemic brain damage [7]. Colored microspheres 15 micrometers in diameter were administered directly into the left cardiac ventricle percutaneously at the end of hypoxia. The rats were killed 24 hours after insult, and brain damage was classified into mild and severe damage groups. In control animals, the blood flow was equally distributed in both hemispheres (Figure 3). Cerebral blood flow distribution of the ligated side decreased significantly, 45% in the mild damage group and 66% in the severe damage group. Thus, in the Levine-Rice model, the more severe the developing brain damage, the greater the percentage difference of blood flow distribution.

### 2.4. Behavioral Tests

We used 3 different learning and memory tasks. Details of these have been described elsewhere [8].

#### 2.4.1. Choice Reaction Time Task (Figure 4(a))

The choice reaction time task represents the first step of cognition and memory and is related to attention and immediate memory retention ability.

Rats were trained for 1 to 2 weeks before the test, in which the rats should press either of 2 levels by varying the correct lever; a cue lamp was randomly lighted above the correct lever. A pellet dispenser released a pellet only when the rat pressed the correct lever. The time between pellet presentation with the cue-lamp on and the correct lever being pressed was defined as choice-reaction time. Parameters were correct responses, incorrect lever pressings, and so forth.

#### 2.4.2. Water Maze Task (Figure 4(b))

The water maze task tests permanent spatial learning ability and reference memory.

The pool was divided into 4 quadrants where one quadrant had a hidden platform in the middle. The test rat was placed in 1 of 3 quadrants (excluding the platform containing one), facing the wall of the pool. Parameters were the time taken to reach the platform (or 120 seconds elapsed), total swimming distance, and swimming speed.

#### 2.4.3. 8-Arm Radial Maze Task (Figure 4(c))

The 8-arm radial maze task is a test for spatial learning ability, which indicates long-term reference memory as well as short-term working memory.

The test rat was placed in a circular plastic ring on the platform at the center of the 8-arm maze. After 1 minute, the ring was lifted, and the rat was allowed to move freely in the maze. The task continued until the rat entered all 8 arms to eat a pellet or until 10 minutes had elapsed. The test was performed every day for 21 days. Test performance was assessed by 3 parameters: correct choices in the initial 8 chosen arms, errors of entry into an already entered arm, and total time.

## 3. Neuroprotective Therapies

Plenty of studies seeking effective neuroprotective therapies have investigated perinatal hypoxic-ischemic encephalopathy. In the last 2 decades, we have also contributed to this field. Some of the findings from our research terms are provided here.

### 3.1. Hypothermia

A protective effect of mild to moderate hypothermia against hypoxic-ischemic brain damage has been shown by a number of studies in adult and neonatal rats. A 3°C reduction in the systemic temperature during 3 h hypoxia provides partial benefit, whereas a 6°C reduction completely protects the brain [9]. A detrimental effect of mild hyperthermia on hypoxicischemic brain damage, in which a 2°C increase exacerbates postischemic brain damage and functional neurologic outcome, has been reported.

**Figure 5:**
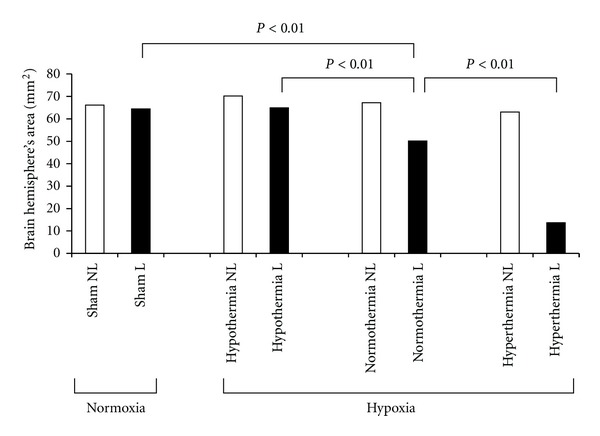
Effect of hypothermia and hyperthermia on histological changes in brain. White bars represent the nonligated side (NL) and black bars represent the ligated side (L) of the brain. Brain hemisphere area was significantly decreased by hyperthermia, whereas it was preserved by hypothermia.

We also performed the hypoxic-ischemic experiments on 7-day-old Wistar rats to see the histological and functional changes in brain development under three different temperature conditions: hypothermia (27°C), normothermia (33°C), and hyperthermia (37°C) [10]. Histologically, hyperthermia during hypoxia-ischemia significantly worsened brain damage, while hypothermia protected against brain damage, compared with normothermic conditions (Figure 5). We also evaluated the influence of temperature conditions on long-lasting neurologic deficits after hypoxia-ischemia in the same animal model. Hypothermia significantly decreased attention deficits in the choice reaction time task and spatial learning deficits in the water maze task. Hyperthermia, however, aggravated those behavioral and memory deficits.

Thus, temperature regulation during hypoxia-ischemia is important, such that hypothermia reduces histological and behavioral deficits after hypoxia-ischemia, but hyperthermia worsens them.

### 3.2. Rehabilitative Training

Rehabilitative manipulations have been demonstrated to improve learning and behavioral disability caused by hypoxia-ischemia in humans as well as in rats [11]. We also tested whether rehabilitative training improves spatial learning impairment in the water maze, after hypoxia-ischemia, in rats. We demonstrated a late-onset, slowly progressive brain damage 5 weeks after hypoxia ischemia [12]. We hypothesized that these progressive histological defects and their related functional impairments could be improved by rehabilitation, since rehabilitative training would increase neurotropic factors in some experimental models. We used 7-day-old Wistar rat models to make hypoxic-ischemic brain damage. Six weeks later, the rats were divided into training and no training groups. We used the water maze task to evaluate spatial learning ability in both groups and then euthanized the rats to evaluate histological changes. Interestingly, the training tasks did not change the hemispheric area of brain damage between the training and no training groups, but swimming distance and speed were significantly improved in the training group. These results suggested that rehabilitative training prevented long-lasting hypoxic-ischemic functional deficits such as learning and memory disability.

### 3.3. Edaravone

**Figure 6:**
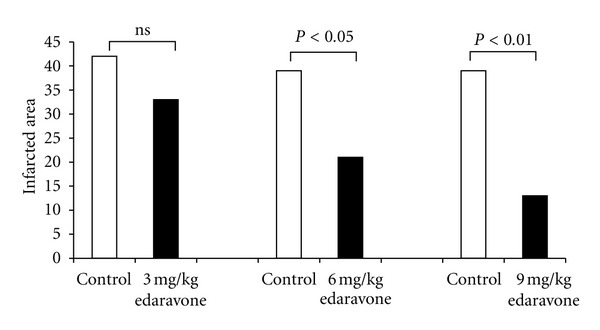
Effect of edaravone on the infracted area. White bars represent controls and black bars represent the edaravone group, where there is a dose-response relationship.

Free radicals are reactive chemicals which are important mediators of cell death and tissue injury after hypoxia-ischemia. Hypoxia-ischemia causes free radical reactions, leading to tissue toxicity, including oxidation of lipid, protein, and polysaccharides. Newborns are at higher risk of oxidative stress and more susceptible to free radical oxidative damage than more mature infants. Thus, we investigated the effect of the free radical scavenger, edaravone, 3-methyl-1-phenyl-2-pyrazolin-5-one, on the development of hypoxic-ischemic brain damage in newborn rats [13]. A Levine-Rice model of 7-day-old rat was made and edaravone was given intraperitoneally. A control group was given saline. Edaravone significantly reduced the brain-damaged area in a dose-response fashion (3, 6, or 9 mg/kg) (Figure 6).

Since edaravone has been approved in Japan for use in patients with cerebral infarction, this is a promising candidate for the treatment of neonatal hypoxic-ischemic encephalopathy. We then performed an experiment to find out whether long-term edaravone treatment is more effective than short-term treatment [14]. With the same Levine-Rice models, edaravone was given after hypoxic-ischemic insult every 24 hours for 2, 5, or 10 consecutive days, and behavioral and histological deficits were evaluated. The 2-day treatment improved learning and memory performance, as well as histological recovery, compared with controls. The 5-day treatment showed histological improvement but no behavioral improvement. However, the 10-day treatment resulted in no improvement in histological or behavioral changes, compared with the controls. These 3 different treatments of edaravone had different impacts on brain histology and behavioral parameters, suggesting that its use is most beneficial for the acute phase after hypoxia-ischemia.

Possible mechanisms by which the free radical scavenger is protective against hypoxic-ischemic brain impairment have also been studied. Hypoxia-ischemia produces free radicals, which initiate lipid peroxidation and maintain generation in a chain reaction, ultimately damaging the cell membrane and causing cell death. So, we studied whether edaravone inhibits lipid peroxidation in hypoxic-ischemic newborn rats [15]. Edaravone significantly decreased lipid peroxidation (thiobarbituric acid reactive substance levels) of the damaged brain hemisphere, compared with saline controls. Furthermore, edaravone significantly decreased the level of nitric oxide metabolites in cerebrospinal fluid at 5 hours after hypoxia. Thus, edaravone improves hypoxic-ischemic brain damage in the developing rat, probably through mechanisms such as transient inhibition of lipid peroxidation and nitric oxide production.

Protective effects of edaravone against hypoxic-ischemic damage were investigated with the aid of an *in vivo* microdialysis technique. We placed a microdialysis probe into the hippocampus and induced hypoxic-ischemic stress in the Levine-Rice rat model. Edaravone or saline was perfused with a spin trap agent and then analyzed by electron paramagnetic resonance spectroscopy. We found that edaravone directly and dose-dependently inhibited lipid free radical formation during the hypoxic-ischemic insult in the neonatal rat brain [16].

Following these experiments with edaravone, we then looked at changes in gene expression caused by hypoxia-ischemia to elucidate molecular events occurring in the brain, as well as the impact of edaravone on gene expression. We performed comprehensive gene expression and gene network analyses using a DNA microarray system. After hypoxia-ischemia alone, there are many upregulated genes, relating to cell death signaling and immune responses, and many downregulated genes reflecting progressive damage, in the contralateral cerebral hemisphere. Comparing these changes, edaravone caused much less gene expression, probably reflecting the protective effect of edaravone against hypoxic-ischemic brain damage [17, 18].

### 3.4. Neurotrophic Factors

One of the new approaches toward the prevention and treatment of hypoxic-ischemic brain damage is neurotrophic factor, which includes nerve growth factor, brain-derived neurotrophic factor, glial cell-derived neurotrophic factor (GDNF), basic fibroblast growth factor, a transforming growth factor group, and a neurotrophin group.

GDNF is a potent neurotrophic peptide and is present in neuronal and nonneuronal cells throughout all regions in the developing brain, suggesting its protective role against hypoxic-ischemic damage.

We first investigated the effects of GDNF in hypoxic-ischemic brain injury in developing rats (Levine-Rice model). Intracerebral injection of 2 or 4 micrograms of GDNF significantly decreased the incidence and severity of brain damage (controls 76–93% versus GDNF 34–64% in 2 micrograms and 7–29% in 4 micrograms, in incidence). This study suggests that GDNF may be protective against perinatal hypoxic-ischemic encephalopathy [19].

We studied the spatial and temporal patterns of GDNF after hypoxia-ischemia in neonatal rat brain and found that significant upregulation of the GDNF protein occurred in a bimodal fashion in the damaged brain hemisphere. The early rise is during the first 3 hours and is probably related to enhanced neuronal release. The second rise is during 72 hours to 1 week and is probably related to progressive astrogliosis after injury [20].

Following the above-mentioned studies, we administered GDNF for hypoxic-ischemic encephalopathy to prevent brain damage in neonatal rats. GDNF is a rather large protein that is impermeable to the blood-brain barrier. For this purpose, we used encapsulated GDNF-secreting cells by using baby hamster kidney cells transfected with human GDNF [21]. The capsule was implanted in the brain at 12 days of life, and hypoxia-ischemia was loaded 2 days after implantation. Compared with the control group, serum GDNF concentrations were significantly elevated and neuronal damage was significantly less in the experimental group [22].

We also investigated the effects of GDNF on long-lasting learning and behavioral changes in the rat model. The encapsulated GDNF was implanted in the 7-day-old Wistar rats, and, 2 days after implantation, a hypoxic-ischemic insult was given. Then several learning tasks were examined, such as the 8-arm radial maze task, the choice-reaction time task, and the water maze task. Improved performance was observed in all three tasks for the GDNF group compared with the control group [23]. Thus, GDNF treatment is effective not only in reducing brain injury, but also in improving learning and memory performances after hypoxic-ischemic insults in the developing rats.

GDNF is also effective in the reduction of the peripheral nerve injury. We produced the Erb's palsy model by transecting the anterior and posterior roots of the left C5–C7 nerves of 7-day-old Wistar rats [24]. The transected edges were kept in contact by each other and nestled by Gelform soaked with 10 microgram GDNF, or saline as control. The behavioral evaluation by foot-fault test was significantly improved by GDNF. As well, the number of anterior horn cells was preserved by GDNF but significantly reduced in saline controls.

### 3.5. Dexamethasone

Corticosteroid therapy has been widely used antenatally to prevent neonatal respiratory distress syndrome, intraventricular hemorrhage, and intestinal perforation, as well as chronic lung disease postnatally. Furthermore, antenatal corticosteroids reduced the risk of periventricular leukomalacia, the most popular cause of neurological complication of the premature infants [25]. Therefore, we performed several studies on the neuroprotective effects of corticosteroid.

**Figure 7:**
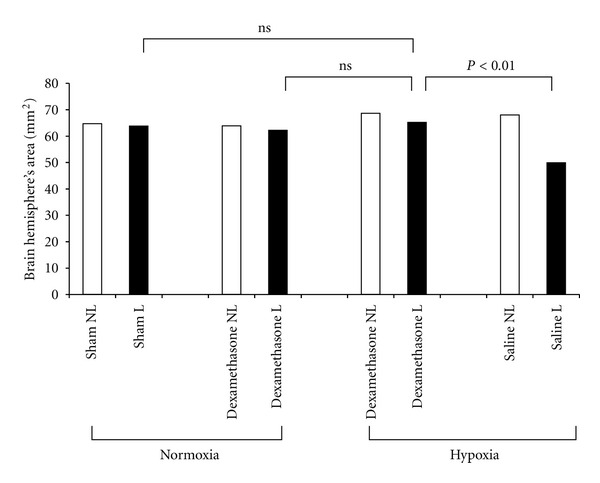
Effect of dexamethasone on brain hemisphere area. White bars represent the nonligated side (NL) and black bars represent the ligated side (L) of the brain. Dexamethasone reversed hypoxic-ischemic brain damage, while dexamethasone under normoxemic condition had no deleterious effects.

Dexamethasone (0.4 mg/kg, intraperitoneally) was injected 4 h before hypoxic-ischemic insult at the postnatal day 7 of the Wistar rat, and learning and memory impairment as well as histological deficits were studied. Dexamethasone treatment completely prevented histological brain damage and significantly improved behavioral and learning abilities (Figure 7). Dexamethasone without hypoxic-ischemic insult caused no adverse effects on learning and memory tests [26].

Similarly, dexamethasone also prevents behavioral and histological damage caused by a combination of lipopolysaccharide and hypoxia-ischemia in neonatal rats [27]. Lipopolysaccharide worsens the hypoxic-ischemic brain damage in a dose-response fashion and in a synergetic manner [28]. Thus, dexamethasone treatment can be a promising candidate for the prevention of inflammation and hypoxia-associated brain damage in clinical settings.

### 3.6. Magnesium

Magnesium is a nonspecific competitive blocker of calcium channel and plays many important roles in maintaining homeostasis of the body. One of its roles is a gating function against calcium influx through the NMDA (N-methyl-D-asparate) receptor-associated ion channels in the brain. Hypoxia-ischemia causes intracellular energy failure that initiates a series of additional mechanisms, such as membrane depolarization, accumulation of excitatory amino acids, and accumulation of cytosolic calcium, which lead to a variety of cascading deleterious effects. We hypothesized that magnesium ion possibly blocks calcium ion influx through the calcium channels and prevents hypoxic-ischemic brain damage.

**Figure 8:**
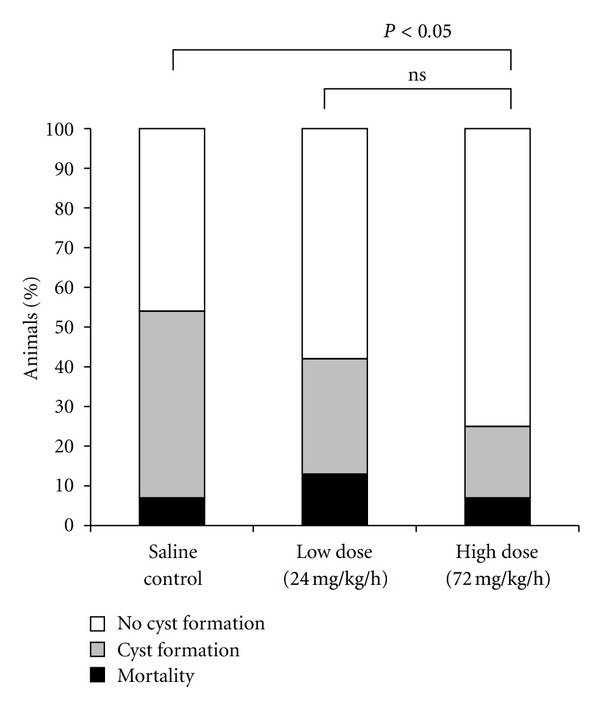
Effect of magnesium sulfate on mortality and brain cyst formation. A positive dose-response relationship existed between magnesium dosage and brain protection.

Using the Levine-Rice neonatal rat model, we first found that prehypoxic treatment of magnesium sulfate ameliorates the severity of brain damage, but posthypoxic treatment deteriorates it. This deleterious effect may be attributable to hypotension caused by high-dose magnesium sulfate, which further worsens cerebral perfusion [29]. From a clinical standpoint, prehypoxic treatment is not practical. So, we studied possible rescue treatment modalities of magnesium sulfate to decrease brain deficits after hypoxia-ischemia. In adult animals, brain magnesium ion concentrations are significantly decreased for several hours or days after ischemia or trauma, and restoration of magnesium ion concentration of the brain improved brain damage. Therefore, we evaluated the effects of long-term (3 days), low-dose magnesium administration on hypoxic-ischemic brain injury in neonatal rats [30, 31]. The serum concentrations of magnesium ion were significantly decreased by hypoxia-ischemia for 3 days in controls. Compared with the controls, magnesium infusion with an osmotic pump restored its concentrations. Brain damage was significantly improved by long-term magnesium administration in a dose-dependent manner, compared with the controls (Figure 8).

**Figure 9:**
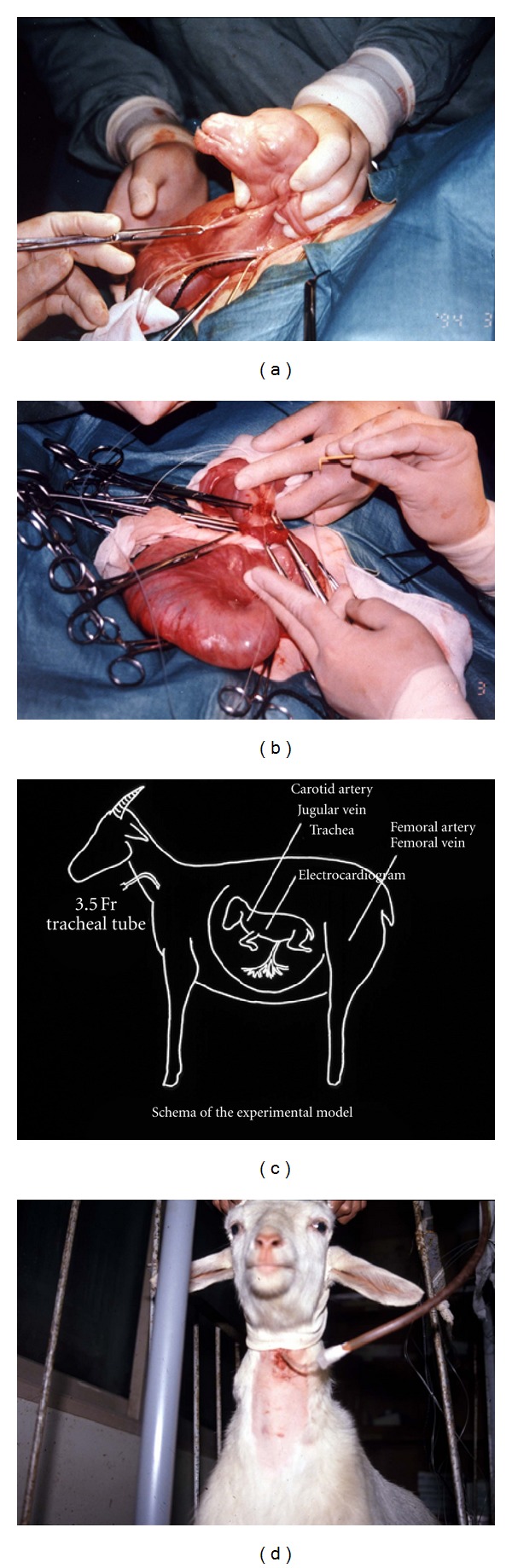
The chronic preparation model using goat fetuses at 0.9 gestation. Under general anesthesia, (a) fetal head and neck was exteriorized, (b) catheters and electrodes were placed, (c) fetus was returned to the uterine cavity and recovered from surgical stresses for 4 days, and (d) hypoxic experiments were performed by adding nitrogen gas through the maternal endotracheal catheter.

**Figure 10:**
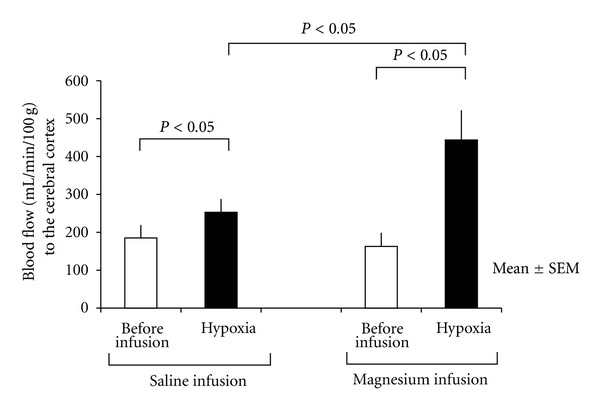
Blood flow changes by hypoxemia alone and hypoxemia under magnesium administration. Hypoxemia significantly increased the cerebral blood flow, and hypoxemia with magnesium further increased cerebral blood flow. Mean ± SE.

Magnesium may indirectly affect brain damage by, for example, increasing blood flow distribution to the brain. To elucidate this possibility, we used a chronically instrumented fetal goat model (Figure 9) and a colored microsphere technique [32]. Magnesium sulfate was directly infused to the fetal cervical vein in a bolus dose of 270 mg/kg followed by 80 mg/kg/h, which is equivalent to the clinical dosage. Hypoxia was induced by adding nitrogen gas to the maternal inhaling air. Fetal PO_2_ significantly decreased from 30 mmHg to 14 mmHg. Hypoxia significantly increased cerebral blood flow, and hypoxia combined with magnesium administration further increased cerebral blood flow (*P* < 0.05) in the cerebral cortex (Figure 10).

### 3.7. Vagal Stimulation

In fetal life, parasympathetic responses are relatively more dominant than sympathetic ones in resting and in hypoxemic conditions, implying their beneficial effects on the fetus. In adults, neuroprotective effects of parasympathetic activation on brain damage have been reported, including inhibition of glutamate release, activation of cholinergic anti-inflammatory pathways to inhibit cytokine release, increase in cerebral blood flow via nitric oxide induction, and enhancement of neurogenesis [33].

**Figure 11:**
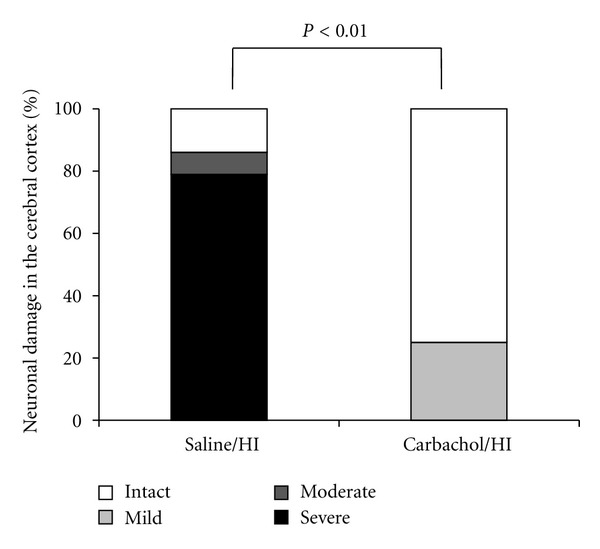
Effect of a parasympathetic agonist, carbachol, on the hypoxic-ischemic brain damage in the neonatal rat. HI is hypoxia-ischemia. Carbachol significantly decreased neuronal damage in the cortex.

We hypothesized that acetylcholine receptor agonists reduce hypoxic-ischemic brain damage in the Levine-Rice model. We injected subcutaneously 0.1 mg/kg of parasympathetic agonist, carbachol (carbamylcholine chloride), or saline as control, just before 2-hour 8% hypoxia-ischemia. The severity of the brain damage was compared between the carbachol group and the saline control. In the cerebral cortex, 25% of the carbachol group showed mild neural damage, and the remaining 75% showed no damage (Figure 11). In contrast, more than 80% of the saline group had severe damage (*P* < 0.05). Thus, vagal stimulation through acetylcholine receptor agonist has a beneficial effect against perinatal hypoxic-ischemic brain damage [34]. This neuroprotective effect is likely related to the effect on microglial activation during hypoxia-ischemia [35]. We also confirmed that, contrary to the neuroprotective effects of acetylcholine receptor agonists, its antagonists worsen hypoxia-ischemia brain damage in neonatal rats [35]. These observations imply that vagal stimulation during hypoxic-ischemic insult is a promising treatment of choice against hypoxic-ischemic neonatal encephalopathy.

### 3.8. Osteopontin

Osteopontin is a glycosylated phosphoprotein and is involved in multiple biological functions such as antiapoptotic processes. Its neuroprotective effect is investigated in the neonatal rat brain after hypoxia-ischemia [36]. First, endogenous expression of osteopontin in the rat brain was significantly decreased during development after birth. Second, osteopontin expression in the brain was significantly increased after hypoxic-ischemia with a peak at 48 hours. Third, osteopontin treatment (both 0.03 and 0.1 microgram) significantly reduced infarct volume compared with the vehicle control. And finally, osteopontin treatment significantly improved some behavioral tests for memory and learning functions. Osteopontin is thought to function through interactions with proteins preferable to apoptosis.

### 3.9. Isoflurane

Some minor insults before the major injurious events may act as preconditioning or tolerance so as to reduce the brain damage. For example, mild degrees of hypoxia, heat stress, and inflammation by lipopolysaccharide are well known for preconditioning activities [37].

Anesthetics may also play a unique role as preconditioning. One of them is isoflurane, which improved neuronal injury induced by oxygen-glucose deprivation *in vitro* [38] and hypoxic-ischemic brain injury in the 7-day-old Levine-Rice model [39]. On the other hand, other reported in the same animal model that isoflurane exerted only a short-term, but not a long-term neuroprotective effect [40]. The differences between these studies may be attributed to varying levels of preconditioning such as duration of isoflurane exposure and recovery time from the prior minor insult to the hypoxia-ischemia.

### 3.10. Granulocyte-Colony Stimulating Factor (G-CSF) and Erythropoietin (EPO)

Similar to the neurotrophic factors, it has long been studied whether blood cell growth factors such as G-CSF and EPO act as neuroprotective in animals as well as in humans.

G-CSF mainly stimulates the development of progenitor cells to neutrophils, but it also has trophic effects on the different cells including neuronal cells. G-CSF also has an anti-inflammatory effect on central nervous system, an antiapoptotic effect on neurons, and a stimulatory effect on neurogenesis [41]. Although the antioxidants data suggest that G-CSF plays a role as a neuroprotectant [41]. In the developing brain, G-CSF also improves hypoxic-ischemic brain damage in the Levine-Rice model. When injected 1 hour before the insult and once per day for 5 days or 10 days thereafter, G-CSF prevented brain atrophy and heart underdevelopment, improved motor and behavioral functions, and improved tests for short-term memory [42].

EPO is an endogenous cytokine that enhances red blood cell production to increase oxygen delivery as a hypoxic physiological response and promotes cell survival via mechanisms of antiapoptotic functions [43]. EPO is neuroprotective in neonatal rat models [44, 45] as well as in clinical settings [46].

### 3.11. Antioxidants

Antioxidants such as dipyridamole, apotransferrin, vitamin E, and N-acetylcysteine are known to have some neuroprotective potentials against oxidative stress including reactive oxygen species and reactive nitrogen species, which are increased during hypoxia and postischemic reperfusion stages. In the developing brain of neonatal rat model, administration of these antioxidants attenuates white matter damage and induces remyelination processes [47, 48].

### 3.12. Stem Cells

Cell therapy containing stem cells has been found to protect neurons from hypoxic-ischemic damage and some degenerative disorders. One of the targets is hypoxic-ischemic brain damage that occurs during labor and delivery and neonatal period.

Mesenchymal stromal cells, bone marrow mesenchymal stem cells, and umbilical cord stem cells have been used in the treatment of neonatal hypoxic-ischemic encephalopathy and show reduction in sensorimotor and cognitive impairments [49–51]. Review articles show that these stem cell therapies are one of the promising options for the treatment of neonatal neurological diseases in the future.

### 3.13. Modulators of K(+)-ATP Channels

K(+)-ATP channels exist on the cell surface and on the inner membrane of the mitochondria [52]. Some controversies exist concerning their role in neuroprotection.

Activation of certain ion channels that are able to attenuate neuronal depolarization may produce neuronal protective effects. One of the candidates is K(+)-ATP channels. Hypoxia reduces intracellular ATP levels that activate K(+)-ATP channels to prevent membrane potential depolarization, leading to neuroprotection [51]. Neuronal hyperpolarization inactivates calcium channels and in turn inhibits calcium-dependent glutamate release, thereby protecting against excitatory neurotoxicity [52].

On the other hand, recent studies also proposed that inhibition of these channels might have protective effects on neuronal survival. For example, in *in vitro* hippocampal slice preparation, K(+)-ATP channel blockers such as tolbutamide and glibenclamide produce neuroprotective effects [53].

## 4. Summary

From the basic animal studies on perinatal hypoxic-ischemic brain damage, we have now obtained some possible candidates for the therapeutic measures against it. They are hypothermia, rehabilitation, free radical scavenger, trophic factors, steroid, calcium channel blocker, vagal stimulation, some antiapoptotic agents, before and after conditioning, antioxidants, cell therapy with stem cells, and modulators of K(+)-ATP channels. Some of them have already been introduced to clinical practice, for example, hypothermia, magnesium, G-CSF, and EPO. Whether combination of these therapies may be more beneficial than any single therapy needs to be clarified.

Hypoxia-ischemia is a complicated condition, in which the cause, severity, magnitude, and deteriorating speed are different in each case. Likewise, each fetus has its own inherent potentials against an hypoxic-ischemic insult, for example, adaptation, preconditioning-tolerance, and intolerance. Therefore, our final goal is an individualized strategy for neuroprotection against perinatal hypoxic-ischemic insult. Further extensive studies are required.

## Figures and Tables

**Figure 4:**
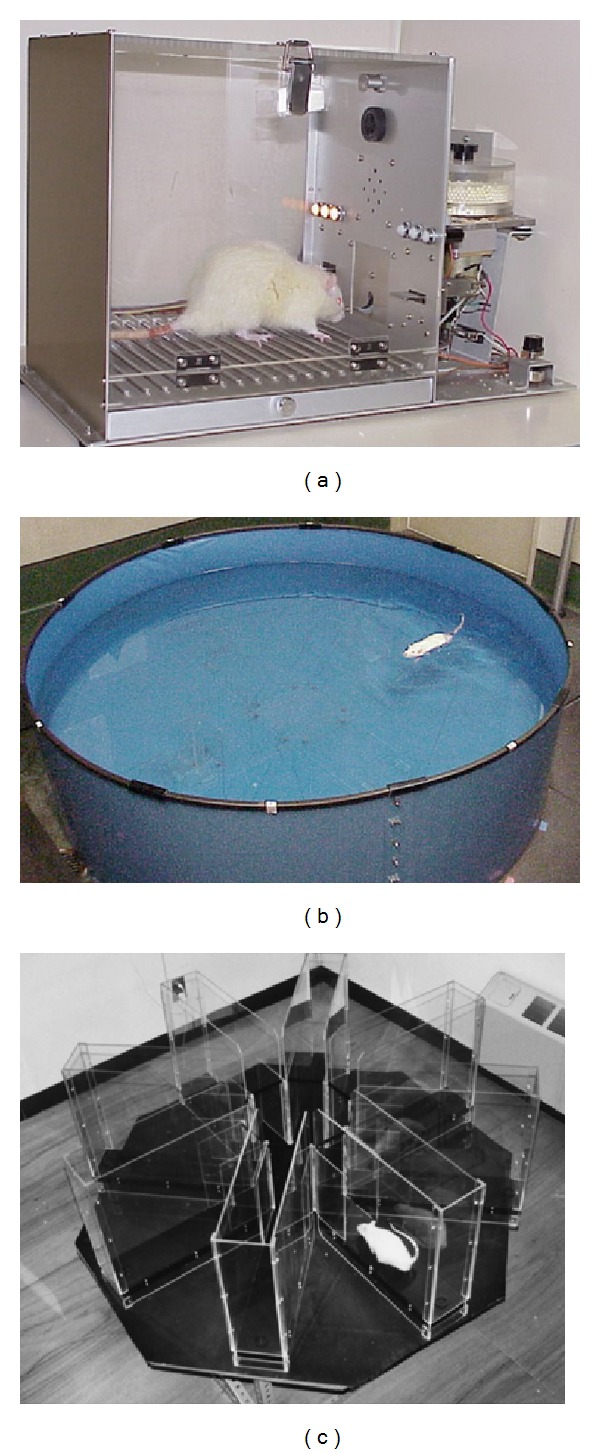
Behavioral tests: (a) choice reaction time task, (b) water maze task, and (c) 8-arm radial maze task.
